# An Educational and Exercise Mobile Phone–Based Intervention to Elicit Electrophysiological Changes and to Improve Psychological Functioning in Adults With Nonspecific Chronic Low Back Pain (BackFit App): Nonrandomized Clinical Trial

**DOI:** 10.2196/29171

**Published:** 2022-03-15

**Authors:** Carolina Sitges, Juan L Terrasa, Nuria García-Dopico, Joan Segur-Ferrer, Olga Velasco-Roldán, Jaume Crespí-Palmer, Ana María González-Roldán, Pedro Montoya

**Affiliations:** 1 Research Institute of Health Sciences (IUNICS) and Balearic Islands Health Research Institute (IdISBa) Department of Psychology University of the Balearic Islands (UIB) Palma Spain; 2 Research Institute of Health Sciences (IUNICS) and Balearic Islands Health Research Institute (IdISBa) Department of Nursing and Physiotherapy University of the Balearic Islands (UIB) Palma Spain; 3 Research Institute of Health Sciences (IUNICS) and Balearic Islands Health Research Institute (IdISBa) University of the Balearic Islands (UIB) Palma Spain

**Keywords:** low back pain, chronic pain, mobile apps, education, exercise, brain, cognition, depression, pain threshold, mHealth, mobile phone

## Abstract

**Background:**

Concomitant psychological and cognitive impairments modulate nociceptive processing and contribute to chronic low back pain (CLBP) maintenance, poorly correlated with radiological findings. Clinical practice guidelines recommend self-management and multidisciplinary educational and exercise-based interventions. However, these recommendations are based on self-reported measurements, which lack evidence of related electrophysiological changes. Furthermore, current mobile health (mHealth) tools for self-management are of low quality and scarce evidence. Thus, it is necessary to increase knowledge on mHealth and electrophysiological changes elicited by current evidence-based interventions.

**Objective:**

The aim of this study is to investigate changes elicited by a self-managed educational and exercise-based 4-week mHealth intervention (*BackFit app*) in electroencephalographic and electrocardiographic activity, pressure pain thresholds (PPTs), pain, disability, and psychological and cognitive functioning in CLBP versus the same intervention in a face-to-face modality.

**Methods:**

A 2-arm parallel nonrandomized clinical trial was conducted at the University of the Balearic Islands (Palma, Spain). A total of 50 patients with nonspecific CLBP were assigned to a self-managed group (23/50, 46%; mean age 45.00, SD 9.13 years; 10/23, 43% men) or a face-to-face group (27/50, 54%; mean age 48.63, SD 7.54 years; 7/27, 26% men). The primary outcomes were electroencephalographic activity (at rest and during a modified version of the Eriksen flanker task) and heart rate variability (at rest), PPTs, and pressure pain intensity ratings. The secondary outcomes were pain, disability, psychological functioning (mood, anxiety, kinesiophobia, pain catastrophizing, and fear-avoidance beliefs), and cognitive performance (percentage of hits and reaction times).

**Results:**

After the intervention, frequency analysis of electroencephalographic resting-state data showed increased beta-2 (16-23 Hz; 0.0020 vs 0.0024; *P*=.02) and beta-3 (23-30 Hz; 0.0013 vs 0.0018; *P*=.03) activity. In addition, source analyses revealed higher power density of beta (16-30 Hz) at the anterior cingulate cortex and alpha (8-12 Hz) at the postcentral gyrus and lower power density of delta (2-4 Hz) at the cuneus and precuneus. Both groups also improved depression (7.74 vs 5.15; *P*=.01), kinesiophobia (22.91 vs 20.87; *P*=.002), activity avoidance (14.49 vs 12.86; *P*<.001), helplessness (6.38 vs 4.74; *P*=.02), fear-avoidance beliefs (35 vs 29.11; *P*=.03), and avoidance of physical activity (12.07 vs 9.28; *P*=.01) scores, but there was an increase in the disability score (6.08 vs 7.5; *P*=.01). No significant differences between the groups or sessions were found in heart rate variability resting-state data, electroencephalographic data from the Eriksen flanker task, PPTs, subjective ratings, or cognitive performance.

**Conclusions:**

Both intervention modalities increased mainly beta activity at rest and improved psychological functioning. Given the limitations of our study, conclusions must be drawn carefully and further research will be needed. Nevertheless, to the best of our knowledge, this is the first study reporting electroencephalographic changes in patients with CLBP after an mHealth intervention.

**Trial Registration:**

ClinicalTrials.gov NCT04576611; https://clinicaltrials.gov/ct2/show/NCT04576611

## Introduction

### Background

Low back pain (LBP) is a highly experienced symptom in the general population and the main cause of disability in industrialized countries [1]. Although its origin is usually unknown and multicausal, numerous factors such as age, sedentary lifestyle and excess weight, psychosocial factors [2], and brain changes related to pain processing [3] favor its maintenance. In addition, symptoms, pathology, and radiological findings are poorly correlated [1], and, consequently, approximately 90%-95% have a nonspecific origin [4]. Moreover, 24%-87% will be recurrent and 50%-70% will be considered chronic LBP (CLBP; symptoms experienced for >12 weeks) [1]. Therefore, effective treatments to prevent and reduce public health expenditure in care and labor concepts [1] and alleviate the symptoms of patients are needed.

Evidence-based clinical guidelines consider physical exercise a key component among the nonpharmacological interventions for patients with LBP, and education has traditionally been used as an integral part of the multidisciplinary treatment, with its importance highlighted in recent decades [5-8]. Specifically, the combination of pain neurophysiology education and therapeutic exercise has shown improvements in pain and functioning in patients with nonspecific CLBP [5-7]. Education must be adapted to individual needs to provide skills to self-manage pain coping [9], include information on the origin and nature of the impairment, and encourage patients to continue with daily life activities [10]. It could be done in person or through brochures, webpages, and mobile apps [11]. Accordingly, the so-called mobile health (mHealth) tools are presented as a cost-effective option for continuously recording type, quantity, and quality of patients’ daily activities using discrete wireless sensors, providing rapid feedback to users and clinicians, supporting telerehabilitation efforts, and decreasing clinic visits [12]. Studies to date using mHealth apps have also shown moderate-quality evidence of reductions in pain and disability in patients with CLBP [13,14].

Regarding physical exercise, a systematic review showed that stretching and strengthening exercises delivered with supervision may improve pain and function, respectively, in patients with CLBP [15]. However, stability exercises seem to be more effective than general exercise and as effective as manual therapy in reducing pain and improving functionality in patients with LBP [16]. Motor control exercises further reduce pain and improve mobility compared with general exercises [17]. Moreover, the performance of hip exercises by patients with CLBP and lumbar instability is more effective than conventional therapy at reducing LBP and levels of disability [18]. Consequently, it seems that trunk stability and resistance exercises are known to be effective interventions to improve the stabilization of the spine [19], but most studies are focused on changes in pain and disability. However, other exercise‐induced changes such as psychological factors (eg, reduced fear, anxiety, and catastrophizing, as well as increased pain self‐efficacy), exercise‐induced analgesia, and functional and structural brain adaptations need to be explored [20].

Therefore, current interventions are inadequate because they are often based on a biomedical model, sidelining the well-documented impairments in central nociceptive processing mechanisms [21]. Evidence of enhanced central sensitization to external painful stimuli is reported in patients with CLBP, manifested by increased subjective pain sensitivity and pain-related structural, functional, and metabolic brain changes, even at rest [22]. A recent review stated that chronic pain mostly changes theta and beta oscillations, particularly in the frontal brain areas [23]. These electrophysiological changes have been recently used as markers for therapeutic efficacy, showing a significant association between pain decrease and a peak theta-alpha frequency increase [24]. Likewise, heart rate variability (HRV) is also postulated as an index of how strongly top-down appraisals, mediated by brain areas (eg, amygdala and medial prefrontal cortex) shape brainstem activity that regulate the heart, providing information about the capacity of an organism to function effectively in a complex environment [25]. A meta-analysis evidenced lower parasympathetic activation in chronic pain, especially in fibromyalgia, compared with healthy controls [26]. Moreover, some studies showed a negative correlation between low-frequency beta rhythms (13-20 Hz), as an index of activity of the somatomotor cortex, and the low-frequency component (0.04-0.15 Hz) of the HRV spectrum, as an index of sympathetic activity [27].

Therefore, it is necessary to clarify the usefulness of these physiological measures in patients with CLBP and the relationship of these measures to concomitant psychological (eg, pain beliefs, catastrophizing, and depression) and cognitive (eg, processing speed, memory, and executive function) alterations that may contribute to the mechanisms of central sensitization [28,29]. Some studies showed that structural brain abnormalities and the cognitive impact of CLBP could be reversed by effective treatments (eg, cognitive behavioral therapy and multidisciplinary pain therapy) [30,31]. However, a recent study revealed significant clinical improvements after pain neuroscience education combined with cognition-targeted motor control training in pain, disability, pressure pain thresholds (PPTs), and physical and mental health without substantial changes in brain gray matter morphologic features [6]. Accordingly, a recent systematic review stated that the effect of exercise therapy on pain and pain modulatory substances (eg, serotonin, norepinephrine, and opioids) or their effects on altering pain-related brain activity areas in patients with musculoskeletal pain remains unclear [32].

### Goal of This Study

The goal of this study is to investigate whether a self-managed program based on education and exercise using a mobile app (*BackFit app*), compared with the same program in a supervised face-to-face modality, produces changes in brain activity, HRV, and pain sensitivity (as primary outcomes) and in self-reported measures of clinical pain, disability, and psychological and cognitive functioning (as secondary outcomes) among patients with nonspecific CLBP. We also explore the relationship between electrophysiological changes in pain sensibility, clinical pain, and disability data and psychological and cognitive functioning.

## Methods

### Study Design

This 2-arm parallel design nonrandomized clinical trial was submitted to ClinicalTrials.gov (NCT04576611). This study is also reported according to the CONSORT-EHEALTH (Consolidated Standards of Reporting Trials of Electronic and Mobile Health Applications and Online Telehealth) statement [33] (Multimedia Appendix 1).

### Participants and Procedure

#### Recruitment

A total of 59 patients with nonspecific CLBP initially participated in this study. First, participants were contacted through email or telephone using a database from a previous study [34]. In addition, information about the study was spread by institutional emailing as well as social media, posters, and leaflets at the University of the Balearic Islands and Sant Joan de Déu Hospital (Palma, Balearic Islands). Potential participants were informed about the aim and development of the study, and if they agreed to participate, they were asked about possible contraindications and exclusion criteria. If participants met the inclusion criteria, they were interviewed at the Research Institute of Health Sciences to collect preintervention data (see *Outcomes* section). Before data collection, participants were given an information sheet, and they signed the informed consent paper form to indicate agreement to participate.

#### Inclusion and Exclusion Criteria

The inclusion criteria were as follows: participants aged 18-59 years with nonspecific CLBP lasting for >12 weeks, of which they have experienced at least three episodes of LBP (lasting for >1 week) [35] during the year before the study, and with access to a smartphone with internet access. The exclusion criteria [36] were as follows: high functional impairment compromising activities such as walking, sitting, or getting up from a chair; pain exacerbated by movement; presence of irradiated pain (sciatic type) or referred pain (pain perceived at a location remote from the site of origin) at lower extremities comprising sensitive or motor alterations; history of spine surgery or spinal or pelvic fracture; hospitalization for serious trauma or injuries due to traffic accidents; history of osteoarthritis in the lower extremities; and history of any systemic diseases with involvement of the locomotor system.

#### Sample Size, Randomization, and Blinding

The sample size was calculated using GRANMO-IMIM [37]. Accepting an *α* risk of .05 and a *β* risk of .20, assuming an estimated common SD of 2.5, and anticipating a dropout rate of 10% in a 2-sided test, 28 participants were needed in each group to recognize as statistically significant a minimum difference of 2 units (in pain intensity measured using a numerical rating scale as an indicator of the therapeutic outcome [38]) between groups, assuming that 2 groups exist.

**Figure 1:**
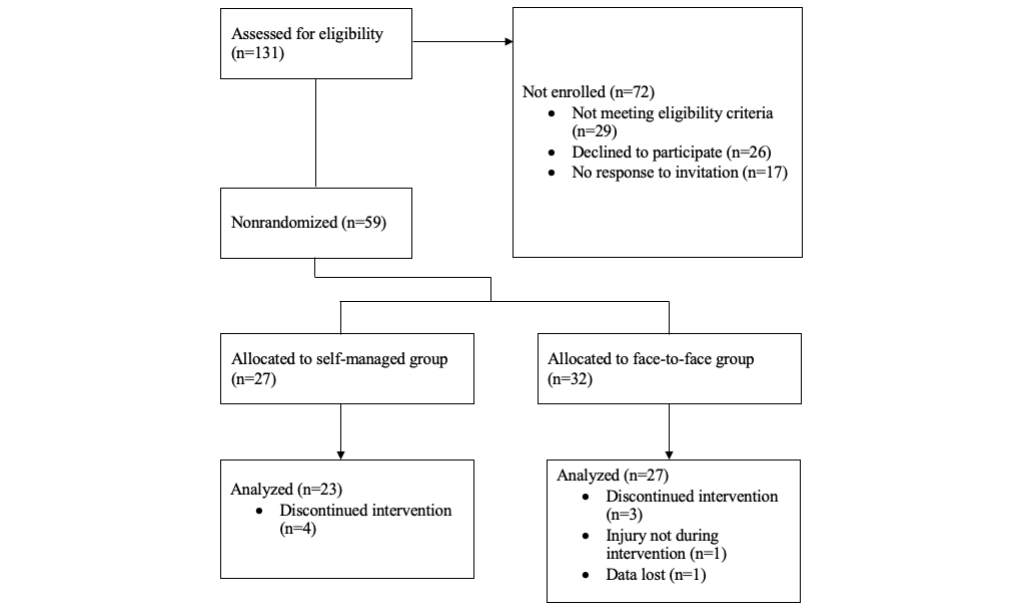
CONSORT (Consolidated Standards of Reporting Trials) flow diagram of the progress of enrollment, intervention allocation, and data analysis.

After compliance to the treatment sessions was checked through the BackFit app, of the 59 participants, we excluded 6 (10%) from the analysis for having undergone fewer than 7 sessions, 1 (2%) because intensity of use was <10 minutes per session in more than one session, 1 (2%) because data were lost (server error), and 1 (2%) for a nonreported previous traffic injury; the remaining 50 (85%) participants were nonrandomly distributed (ie, considering their preferences to promote treatment adherence) into two groups of a 4-week educational and exercise program (total of 8 sessions of approximately 50 minutes’ duration; Figure 1): (1) face-to-face group, supervised by a trained professional (with a degree in physiotherapy and science in physical activity and sport) in small groups (maximum of 4 participants) or individually (as an exception), or (2) self-managed at home using the BackFit app (version 1.0.7 for iOS and version 1.1.5 for Android). The researchers tasked with analyzing data were not involved in the intervention protocol administration, and they were also blinded to treatment allocation.

### Intervention

#### Protocol

**Figure 2:**
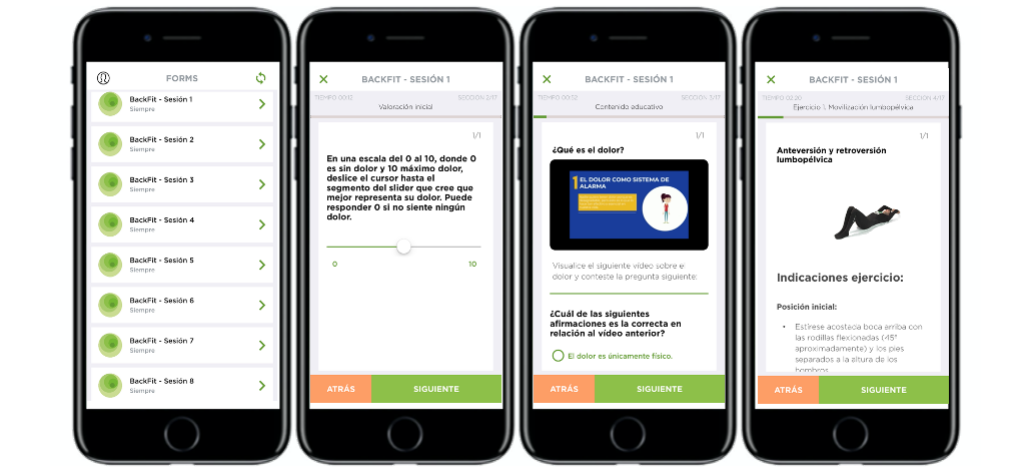
Screenshots from the BackFit app showing examples of the intervention protocol (ie, session, pain rating scale, educational video, and exercise).

All participants had to perform the same intervention protocol twice a week for 4 consecutive weeks, completing up to 8 sessions. Each session consisted of the following: (1) viewing a pain education video <4 minutes in duration [39] (which included information about the neurophysiology of pain; the relationship among pain, exercise, and emotions; causes; risk factors; treatments for LBP [physical activity, cognitive behavioral therapy, and self-massage]; health habits; and self-management for chronic pain); (2) answering a question about the video to ensure that participants have watched it; and (3) performing an approximately 50-minute exercise session based on the recommendations of the American College of Sports Medicine [40], the European guidelines for the management of nonspecific CLBP [41], and some previous studies [42,43], which consisted of muscle strength exercises, motor control, relaxation routines, flexibility, and self-massage, guided by the supervisor or supported by a video showing the exercises and a detailed written description of how to perform them correctly (Figure 2). All participants also rated their actual clinical pain using a slider (0-10) before and after each session and their perceived exertion using a Borg Rating of Perceived Exertion Scale (0-10) after each exercise. The researchers provided a user account (email) and a password to all participants and helped them to configure the BackFit app on their own mobile device. All participants were informed in advance about the weekday on which the session was scheduled (and they were also reminded through notifications from the app or WhatsApp messages delivered to their mobile phone). If a participant could not perform the session on the assigned day, it was rescheduled for another day, always keeping in mind a rest period of 1-4 days between sessions. If participants were in the face-to-face intervention group, they met with the supervisor twice a week at the University of the Balearic Islands (in a room equipped for physical exercise). If participants were in the self-managed intervention group, they received the material (a rubber massage ball [60 kg/cm^2^ in density and 6 cm in diameter] and a foam roller (60 kg/cm^2^ in density, 90 cm in length, and 10 cm in diameter]) for use when performing the exercises at home. After the 4-week intervention, participants returned the material and met with the researchers again to enable collection of the outcome measures described in the *Sociodemographic and Clinical Data* section (after the intervention). The BackFit app had been previously tested among the researchers and by a regulatory agency (the Andalusian Agency for Healthcare Quality) [44], to acknowledge its quality and safety.

#### Sociodemographic and Clinical Data

Sociodemographic and clinical data (using a semistructured interview, height and weight measuring scales, and a digital tensiometer [OMRON M3; OMRON Healthcare]), as well as clinical pain intensity ratings (using a digital slider integrated into the BackFit app) were collected.

### Outcomes

All outcome measures, whether primary or secondary, were collected before and after the intervention.

#### Primary Outcomes

##### Electrophysiological Data Acquisition, Preprocessing, and Analysis

Electroencephalographic signals were continuously recorded for 5 minutes in the eyes-open resting state and during the performance of a cognitive task in an acoustically attenuated room using a QuickAmp amplifier (Brain Products GmbH) at 1000 Hz sampling rate from 29 silver or silver chloride scalp electrodes placed according to the 10-20 System of Electrode Placement. Active electrodes were recorded against an average reference. A ground electrode was located at the AFz position. An electro-oculogram channel was obtained by placing an electrode above the left eye and another below the same eye. An electrocardiogram (ECG) channel was also obtained by placing an electrode at both wrists. Electrode impedances were kept below 10 k*Ω*.

During electroencephalography (EEG) data preprocessing performed with BrainVision Analyzer software (version 1.05; Brain Products GmbH), signals were segmented in epochs of 1000 ms (for resting-state data) or in epochs of 600 ms (−100 to 500 ms, relative to the stimulus onset for cognitive task data) and digitally filtered (high-pass filter at 0.10 Hz, low-pass filter at 30 Hz, and notch filter at 50 Hz). We corrected eye movement artifacts using the Gratton and Coles algorithm [45]. Next, an artifact rejection protocol with the following criteria was applied: maximal allowed voltage step per sampling point=100 mV, minimal allowed amplitude=−100 mV, maximal allowed amplitude=100 mV, and maximal allowed absolute difference in the epoch=100 mV.

Regarding EEG resting-state data, frequency power densities at delta (2-4 Hz), theta (4-8 Hz), alpha (8-12 Hz), beta-1 (12-16 Hz), beta-2 (16-23 Hz), and beta-3 (23-30 Hz) were computed by using the fast Fourier transformation obtained from each artifact-free EEG epoch. A source localization of the frequency bands was also performed by using low-resolution electromagnetic tomography analysis [46]. Electrode coordinates were based on an extended 10-20 system template and expressed as Talairach space coordinates. Subsequently, current source densities of all frequency bands during the resting state were estimated. To reduce interparticipant variability, spectra values were normalized at each voxel. Furthermore, a statistical nonparametric mapping randomization test was used to correct critical probability threshold values for multiple comparisons. A total of 5000 permutations were used to determine the significance of each randomization test. Subsequently, the standardized low-resolution electromagnetic tomography analysis images at each frequency band were generated by comparing the current density after the intervention with that before the intervention for all participants (paired sample 2-tailed *t* tests) and by comparing the current density in the face-to-face group with that in the self-managed group for each session separately (independent sample 2-tailed *t* tests). Voxels with significant session or group differences (*P*<.05) were located using the Montreal Neurological Institute and Hospital coordinates and Brodmann areas (BAs).

Regarding the ECG data, resting-state raw signals were offline filtered (bandpass filter 0.5-30 Hz) and hand corrected for artifacts such as missed, erroneous, or ectopic beats by using QRSTool software [47]. Next, interbeat interval values were extracted and several HRV metrics of the time and frequency domain were computed using Kubios HRV Standard software (version 3.3.1) [48]. In the time domain, mean heart rate (HR), SD of the normal-to-normal (R-R) intervals (SDNN), and the root mean square of the successive differences (RMSSD) were calculated. In the frequency domain, the power in ms^2^ of the very low frequency (VLF; 0-0.04 Hz), the low frequency (LF; 0.04-0.15 Hz), and the high frequency (HF; 0.15-0.4 Hz) were calculated. All these metrics, except for the mean HR, were transformed using a Napierian logarithm scale before the statistical analyses.

Regarding EEG registration during the cognitive task data, a nonparametric cluster-based permutation test (CBPT), which allows for testing group differences in high-dimensional neural data while it deals with the multiple-comparison problem [49], was performed by using FieldTrip toolbox [50] running in MATLAB R2018b. We used the data recorded by the 29 scalp electrodes and a time window from 0 to 500 ms after the congruent and incongruent stimulus presentations for CBPT. For every sample (electrode×millisecond), the face-to-face and self-managed groups were compared in each condition and session by means of an independent sample *t* test (2-tailed). In addition, pre- and postintervention data were compared separately for each group and in each condition by means of a dependent sample *t* test (2-tailed). Samples with *t* values higher than the critical level (*P*<.05) were selected and clustered by temporal and spatial adjacency. Next, *t* values within each cluster were summed to calculate the cluster-level statistics. These observed cluster-level statistics were evaluated through a nonparametric permutation test. The permutations were created by randomly assigning labels and running the test 1000 times, retrieving the maximum cluster statistic every time. Only if the observed cluster-level statistics from the real data were >95% of the maximum cluster statistics in the permutation distribution (Monte Carlo significance probability) were they considered significant.

##### Pain Sensitivity

To assess PPTs, we used a digital algometer (FPIX 50; Wagner Instruments) at an individual unilateral low back location (spinal erector muscle, 2 cm from the spine at the most painful point) and at the forefinger (control) 3 consecutive times in counterbalanced order (maximum pressure of 5 kg/cm^2^). Subjective pressure pain intensity ratings were measured using a visual analog scale (0-10). The average of 3 measurements of both variables was used for the statistical analysis. Algometry was always conducted by the same researcher (OVR).

#### Secondary Outcomes

##### Self-reported Data

Handedness, physical disability, mood, anxiety, fear of movement, pain catastrophizing, and fear-avoidance beliefs were self-assessed on paper using the Spanish versions of the Edinburgh Handedness Inventory [51], the Oswestry Disability Index (ODI) [52], the Profile of Mood States (POMS) [53], the State–Trait Anxiety Inventory [54], the Tampa Scale for Kinesiophobia (TSK-11) [55], the Pain Catastrophizing Scale (PCS) [56], and the Fear-Avoidance Beliefs Questionnaire (FABQ) [57], respectively.

##### Cognitive Performance

A modified computerized version of the Eriksen flanker task [58], frequently and successfully used as a measure of interference control, was used. It included 288 trials, presented in 6 blocks of 48 stimuli (ie, 5 arrows) with an intertrial interval of 600-800 ms. Half of the trials were congruent (ie, the middle arrow points in the same direction as the flankers) and the other half were incongruent (ie, the middle arrow points in a direction opposite to that of the flankers). At each trial, participants were asked to indicate the direction of the middle arrow as quickly and as accurately as possible by pressing the left or right button on a 2-key device. We analyzed cognitive performance as accuracy (percentage of hits) and reaction times (RTs; in ms).

### Statistical Analysis

#### Effects of the Intervention

To investigate the effects of the intervention and the group differences, 2-way analyses of variance with repeated measures were performed using *group* (face-to-face group and self-managed group) as the between-participant factor and *session* (before and after the intervention) as the within-participant factor in sociodemographic, clinical, and self-reported data; in PPTs and pressure pain ratings in both body locations (spinal erector muscle and forefinger); and in each HRV metric in the time (HR, SDNN, and RMSSD) and frequency domain (VLF, LF, and HF), with *condition* (congruent and incongruent) as the within-participant factor in cognitive performance (percentage of hits and RTs) and *channels* (29 electrodes) in each frequency band (delta, theta, alpha, beta-1, beta-2, and beta-3). The chi-square test was used for testing the groups’ gender distribution.

We also calculated pre–post differences in each group and ran a bivariate Pearson correlation analysis only among the variables that showed significant differences in the previous analysis.

All significant results are presented with the original df, the *P* values, and the partial eta squared (*η*_p_^2^) parameters. Except for the CBPT and source localization analysis, all statistical analyses were performed using SPSS for Mac (version 25.0; IBM Corp).

#### Data Exclusion

In all, 10 and 13 outlier values (>3 times the IQR) were excluded from the self-reported (ODI, POMS, PCS, and TSK-11) data analysis and accuracy data analysis, respectively.

### Ethics Approval

This study was conducted according to the Declaration of Helsinki and approved by the research ethics committee of the Balearic Islands (IB 3186/16 PI).

## Results

### Sociodemographic and Clinical Data

As shown in Table 1, both groups were comparable in terms of gender, age, anthropometrics (BMI, waist-to-height ratio, and waist-to-hip ratio), blood pressure (systolic and diastolic), pain duration, handedness, and anxiety (state and trait). Both groups were also comparable in all preintervention measures.

**Table 1:** Sociodemographic, clinical, and self-reported data of participants (N=50).

Characteristics		Before the intervention			After the intervention				*P* value
		Face-to-face group (n=27)	Self-managed group (n=23)	Face-to-face group (n=27)		Self-managed group (n=23)			
Sex (male), n (%)		7 (26)	10 (43)	N/A^a^		N/A		.19^b^	
Age (years), mean (SD)		48.63 (7.54)	45.00 (9.13)	N/A		N/A		.13^c^	
BMI, mean (SD)		0.43 (0.09)	0.41 (0.07)	N/A		N/A		.62^c^	
WHtR^d^, mean (SD)		0.55 (0.08)	0.53 (0.06)	N/A		N/A		.43^c^	
WHR^e^, mean (SD)		1.12 (0.12)	1.14 (0.11)	N/A		N/A		.60^c^	
Pain duration (years), mean (SD)		11.81 (7.47)	8.06 (8.74)	N/A		N/A		.16^c^	
EHI^f^ (10-50), mean (SD)		18.05 (5.06)	18.22 (3.83)	N/A		N/A		.91^c^	
Systolic BP^g^, mean (SD)		112.67 (12.58)	115.30 (14.79)	N/A		N/A		.12^c^	
Diastolic BP, mean (SD)		77.61 (8.62)	76.16 (9.74)	N/A		N/A		.92^c^	
Pain intensity (0-10), mean (SD)		2.87 (2.27)	3.57 (2.50)	2.67 (2.36)		3.83 (2.20)		.93	
ODI^h^ (0-100, %), mean (SD)		6.15 (5.35)	6.01 (3.92)	7.85 (6.22)		7.15 (5.66)		.01^i^	
**POMS^j^, mean (SD)**									
	Tension or anxiety (0-36)	9.96 (7.47)	8.83 (5.94)	7.46 (3.67)			8.00 (5.89)	.06	
	Anger or hostility (0-48)	11.46 (8.48)	9.11 (7.32)	7.96 (4.66)			8.78 (5.29)	.10	
	Vigor or activity (0-32)	15.87 (4.66)	14.28 (5.13)	16.04 (4.75)			16.33 (4.52)	.09	
	Fatigue or inertia (0-28)	9.38 (7.93)	10.15 (7.14)	8.03 (5.91)			8.94 (5.71)	.12	
	Depression or dejection (0-60)	9.26 (11.10)	6.22 (6.65)	5.12 (5.95)			5.17 (5.68)	.01^i^	
	Confusion or bewilderment (0-28)	6.28 (5.18)	5.00 (4.51)	5.04 (4.39)			5.06 (3.57)	.23	
**STAI^k^, mean (SD)**									
	State (0-30)	15.51 (8.76)	13.75 (7.29)	15.49 (8.07)		14.96 (10.12)		.53	
	Trait (0-30)	19.68 (8.02)	20.15 (8.83)	N/A		N/A		.84^c^	
**TSK-11^l^ (11-44), mean (SD)**		23.14 (3.61)	22.68 (3.59)	20.41 (3.45)		21.32 (3.50)		.002^i^	
	Activity avoidance (7-28)	15.09 (2.27)	13.89 (2.31)	12.67 (2.21)		13.05 (2.39)		<.001^i^	
	Harm (4-16)	8.06 (1.69)	8.79 (1.84)	7.74 (1.68)		8.26 (1.52)		.21	
**PCS^m^ (0-52), mean (SD)**		12.21 (8.96)	15.35 (8.53)	12.29 (10.33)		11.05 (6.74)		.19	
	Rumination (0-16)	3.75 (3.77)	4.57 (3.20)	4.29 (3.69)		4.05 (3.39)		.98	
	Helplessness (0-24)	5.83 (4.36)	6.92 (4.14)	4.92 (4.60)		4.55 (3.10)		.02^i^	
	Magnification (0-18)	2.62 (1.64)	3.55 (2.33)	3.08 (2.60)		2.45 (1.64)		.39	
**FABQ^n^ (0-96), mean (SD)**		34.75 (24.08)	35.25 (21.33)	31.96 (19.72)		26.25 (14.54)		.03^i^	
	Avoidance of physical activity (0-24)	12.04 (4.93)	12.10 (6.36)	11.71 (6.52)		6.85 (4.44)		.01^i^	
	Avoidance of work (0-42)	15.79 (12.25)	16.90 (11.72)	13.96 (10.77)		13.65 (9.25)		.10	

^a^N/A: not applicable.

^b^Chi-square test.

^c^Both groups were comparable in terms of gender, age, anthropometrics (BMI, waist-to-height ratio, and waist-to-hip ratio), systolic and diastolic blood pressure, pain duration, handedness, and anxiety trait.

^d^WHtR: waist-to-height ratio.

^e^WHR: waist-to-hip ratio.

^f^EHI: Edinburgh Handedness Inventory.

^g^BP: blood pressure.

^h^ODI: Oswestry Disability Index.

^i^Both groups showed decreased depression, kinesiophobia (and activity avoidance), helplessness, and fear-avoidance beliefs (and avoidance of physical activity), as well as increased disability after the intervention. No significant differences between the groups were found in any of these data.

^j^POMS: Profile of Mood States.

^k^STAI: State–Trait Anxiety Inventory.

^l^TSK-11: Tampa Scale for Kinesiophobia.

^m^PCS: Pain Catastrophizing Scale.

^n^FABQ: Fear-Avoidance Beliefs Questionnaire.

### Primary Outcomes

#### EEG and ECG Resting-State Data

Regarding the frequency power density of the EEG resting-state data analysis, no differences between the groups were found at delta, theta, alpha, beta-1, beta-2, or beta-3 (results not shown). We only found main effect of *session* at beta-2 (*F*_1,47_=5.178; *P*=.02; *η*_p_^2^=0.099) and at beta-3 (*F*_1,47_=4.701; *P*=.03; *η*_p_^2^=0.091), showing increased beta-2 (mean 0.0020, SD 0.0013 µV^2^/Hz vs mean 0.0024, SD 0.0017 µV^2^/Hz) and beta-3 (mean 0.0013, SD 0.0010 µV^2^/Hz vs mean 0.0018, SD 0.0019 µV^2^/Hz) after the intervention in comparison with before the intervention.

**Figure 3:**
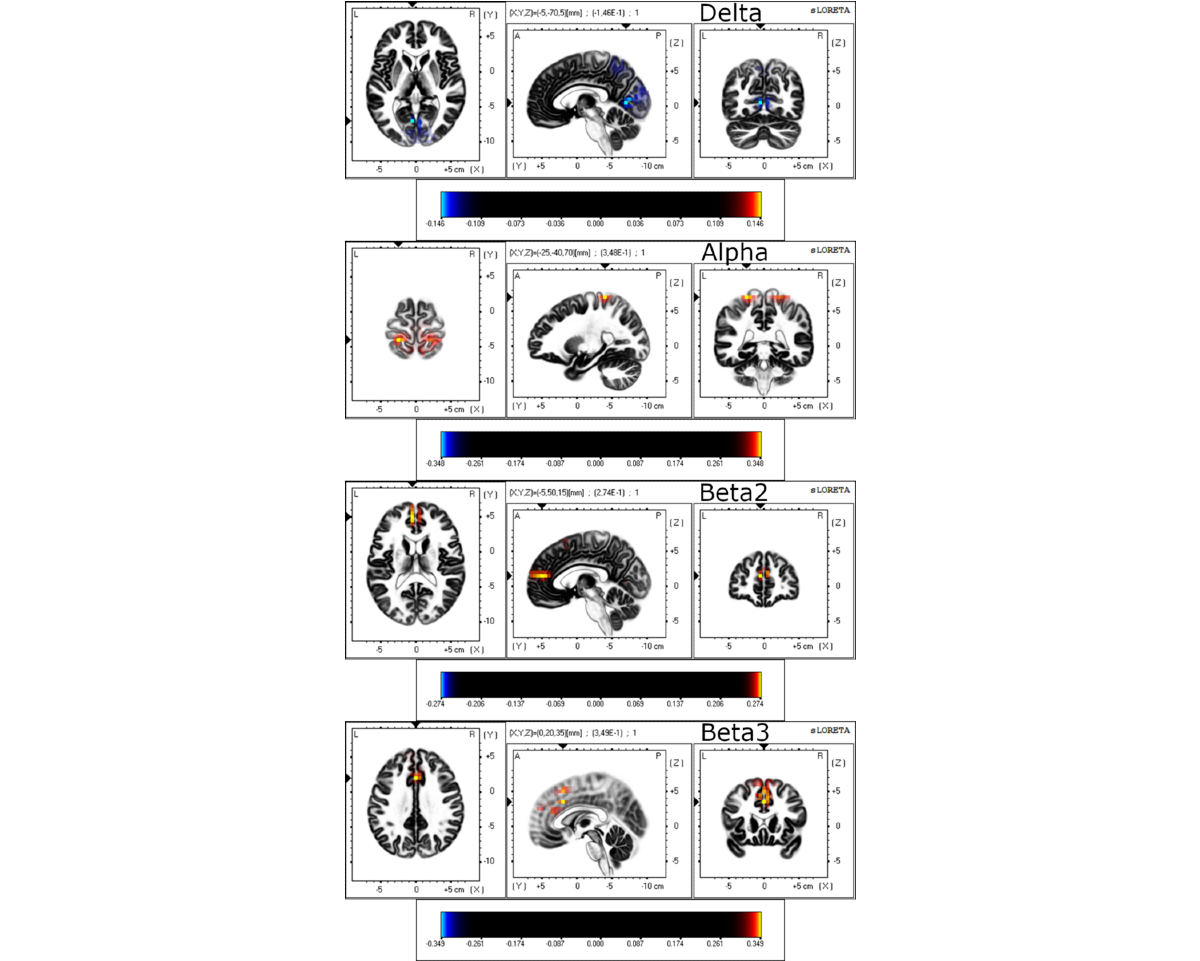
Standardized low-resolution electromagnetic tomography analysis (sLORETA) results for 3 orthogonal brain slices (horizontal, sagittal, and coronal) of delta, alpha, beta-2, and beta-3 frequency bands in all participants. Yellow-red voxels represent increased (*P*<.05) current density after the session compared with before the session. Blue voxels represent decreased (*P*<.05) current density after the session compared with before the session.

Differences between before and after the intervention on statistical maps of source analyses in all participants are displayed in Table 2 and Figure 3. These analyses (paired sample 2-tailed *t* tests) revealed a significant lower current density of delta activity after the intervention compared with before the intervention in the occipital lobe areas at the cuneus (BA30 and BA18) and the middle occipital gyrus (BA18), as well as in the parietal lobe areas at the precuneus (BA7). Moreover, a significant higher current density of alpha activity after the intervention compared with before the intervention was found at the postcentral gyrus (BA2, BA3, BA5, and BA7). Finally, a significant higher current density of beta-2 and beta-3 activity after the intervention compared with before the intervention was found at the anterior cingulate cortex (ACC; BA32 and BA24) and at the medial frontal gyrus (BA10, BA9, BA8, and BA6). No significant differences between the groups before and after the intervention (independent sample 2-tailed *t* tests) were found.

Regarding ECG data, no differences between the groups or sessions were found in HR, SDNN, or RMSSD. No differences between the groups or sessions were found in VLF, LF, or HF (Multimedia Appendix 1).

**Table 2:** Summary of significant results^a^ from whole-brain standardized low-resolution electromagnetic tomography analysis comparisons between before the intervention and after the intervention for delta, alpha, beta-2, and beta-3 frequency bands in all participants.

Lobe and region			BA^b^	X^c^	Y^c^	Z^c^
**Delta (after the intervention<before the intervention)**						
	**Occipital**					
		Cuneus	30	–5	–70	5
		Cuneus	18	0	–75	10
		Middle occipital gyrus	18	25	–90	15
	**Parietal**					
		Precuneus	7	0	–60	55
**Alpha** **(** **after the intervention** **>** **before the intervention** **)**						
	**Parietal**					
		Postcentral gyrus	2	–25	–40	70
		Postcentral gyrus	3	–20	–40	70
		Postcentral gyrus	5	–25	–45	70
		Postcentral gyrus	7	5	–65	65
**Beta-2 (after the intervention** **>** **before the intervention)**						
	**Limbic**					
		Anterior cingulate	32	0	35	20
	**Frontal**					
		Medial frontal gyrus	10	–5	50	15
		Medial frontal gyrus	9	5	50	20
**Beta-3** **(after the intervention** **>** **before the intervention)**						
	**Limbic**					
		Anterior cingulate	32	0	20	35
		Anterior cingulate	24	0	30	25
	**Frontal**					
		Medial frontal gyrus	8	0	20	50
		Medial frontal gyrus	6	–5	15	50

^a^Significant (*P*<.05) regions are indicated with the name of Brodmann area and Montreal Neurological Institute and Hospital coordinates of the higher statistical 2-tailed threshold voxel.

^b^BA: Brodmann area.

^c^Montreal Neurological Institute and Hospital coordinates.

#### EEG Flanker Task Data

The CBPT revealed no differences between the groups or sessions in the EEG response to the congruent or incongruent conditions (results not shown).

#### Pain Sensitivity

No significant differences between the groups or sessions were found in PPTs either in pressure pain intensity ratings at the spinal erector muscle or the forefinger (Multimedia Appendix 1).

### Secondary Outcomes

#### Self-reported Data

Both groups showed decreased depression, kinesiophobia (and activity avoidance), helplessness, and fear-avoidance beliefs (and physical activity avoidance), as well as increased disability after the intervention (Table 1). However, most of our participants showed a minimal disability at baseline (48/50, 96% showed a score of 0-20 measured using the ODI) and did not show a minimal clinical difference after the intervention (45/50, 91% showed a score difference between before the intervention and after the intervention of <10 points).

#### Cognitive Performance

The Eriksen flanker task involved a low level of difficulty (mean overall hit rate 98.93%, SD 0.14%), and no significant main effects in the percentage of hits among the groups, sessions, conditions, or interaction effects were found (results not shown). No significant main effects in RTs among the groups, sessions, or interaction effects were found (results not shown). We only found an expected significant main effect of *condition* (*F*_1,47_=47.255; *P*<.001; *η*_p_^2^=0.501), showing slower RTs in the incongruent trials than in the congruent trials (mean 495.28, SD 76.11 ms vs mean 481.08, SD 84.67 ms).

#### Correlational Data

We only computed a bivariate Pearson correlation analysis of the pre–post differences in psychological outcomes (ODI, POMS depression and dejection scale, TSK-11 total, TSK-11 activity avoidance scale, PCS helplessness scale, FABQ total, and FABQ avoidance of physical activity scale) and EEG resting-state data (delta at the cuneus [BA30], alpha at the postcentral gyrus [BA2], and beta-2 and beta-3 at the ACC [BA32]) in all participants. After applying multiple comparison corrections, no significant correlations were found among these variables.

## Discussion

### Principal Findings

Both groups showed an increase in beta-2 and beta-3 in EEG resting-state data after the intervention. Source localization data analysis also showed a significant higher current density of beta-2 and beta-3 mainly located at the ACC after the intervention, as well as a higher current density of alpha mainly located at the postcentral gyrus and a significant lower current density of delta frequency located at the cuneus and precuneus. Several studies demonstrate that alpha and beta oscillations are related to feedback (top-down) brain signaling or contextual (ie, cognitive, emotional, or motivational) processing of pain [23]. Moreover, changes in brain activation and connectivity during rest in patients with chronic pain are often circumscribed to brain regions related to pain perception [22,59], which would involve the brain regions showing changes in our study (ie, postcentral gyrus and ACC). In this regard, beta band activity in somatosensory areas is increased during motor planning or during maintenance of steady posture, reflecting top-down control of behavior [60]. In contrast, the amplitude of alpha oscillations (before a phasic painful stimulation) over the sensorimotor cortex is negatively correlated with pain perception [23]. Furthermore, patients with chronic pain are characterized by a general trend toward increased power at lower EEG frequencies [61]. Indeed, delta oscillations seem to increase in states of motivational urges triggered by biological rewards and danger (eg, sustained pain) [62], and our intervention has succeeded in reducing the current density of this frequency band. Therefore, our study suggests that both intervention modalities, based on education and exercise, were able to induce neurophysiological changes, mainly in beta-2 and beta-3 frequency bands located at the ACC in patients with CLBP. However, these results should be interpreted carefully because the absence of a control group does not allow establishing a cause-and-effect relationship because some confounding variables (eg, regression to the mean) may be influencing these postintervention effects.

Nevertheless, no significant differences between the groups or sessions in HRV resting-state measures were found. Previous research stated that self-reported pain and RMSSD were inversely associated in healthy individuals but not in chronic pain, concluding that this vagal tone measure is disturbed [63]. Another study conducted on patients with CLBP showed a negative correlation between HRV and physical disability but not with pain [64]. A previous study conducted on patients with CLBP showed that a 3-month yoga intervention decreased self-reported worst pain in the past 2 weeks, LF-HRV, and rate of respiration and increased HF-HRV and PNN50 (indicating parasympathetic activity; PNN50 is the proportion of NN50 divided by the total number of normal-to-normal [R-R] intervals, and NN50 is the number of times successive heartbeat intervals exceed 50 ms) compared with standard medical care [65]. Thus, perhaps the duration of the intervention program or the intensity of the exercises was not sufficient to elicit significant changes in HRV resting-state data.

In addition, the self-managed intervention was as effective as the face-to-face intervention in improving depression, kinesiophobia (plus activity avoidance), helplessness, and fear-avoidance beliefs (plus physical activity avoidance). However, both modalities failed to reduce pain and disability and increase PPTs. In this regard, a previous study found improvements not only in health-related quality of life (mental and physical well-being), kinesiophobia, and hypervigilance, but also in pain sensitivity and disability in patients with CLBP after a 12-week intervention combining pain neuroscience education and cognition-targeted motor control training [6]. However, pain-reducing effect sizes were small to medium (ie, an increase in PPTs of >15% and a decrease in pain scores measured using a numerical rating scale) and failed to find brain morphologic changes. In contrast, we found psychological improvements accompanied by changes in EEG resting-state data, but we failed to find enhancements in pain and disability self-reported scores. Notably, our intervention was of shorter duration and the baseline pain and disability scores of our participants were clearly lower than those reported in previous studies, hindering the possibility of finding significant changes after the intervention and compromising the external validity of our study. Similarly, a 4-week program with 8 sessions, using a self-managed website (including cognitive behavioral therapy as well as motivational and wellness activity advice), evidenced clinically significant decreases in depression, anxiety, and stress, as well as greater use of positive coping strategies but no improvements in self-efficacy, self-reported pain, or physical functioning versus the control group [66]. Thus, it is possible that the duration of the intervention was not sufficient to elicit self-reported changes in pain and disability scores. Nevertheless, the presence of significant electrophysiological changes without improvements in self-reported pain and disability scores challenges the clinical relevance of our results. In addition, the inconsistences found between our research and previously reported studies highlight the need for further research in this field.

Regarding the modality of the intervention, a recent meta-analysis concluded that mHealth-based self-managed programs revealed better immediate effects on pain and disability than web-health–based programs, with better immediate effects on pain but not on disability for programs with durations of ≤8 weeks [13]. High-quality clinical practice guidelines for the noninvasive management of CLBP recommend pain education and physical exercise, considering patient preferences, with a maximum frequency and duration of 8 sessions over 12 weeks [67]. Updated evidence on rehabilitation for chronic pain showed that all exercise modalities seem to be effective compared with minimal, passive, or conservative exercise modalities or no intervention; therefore, there is no evidence indicating which duration, intensity, and training parameters are the most effective [68]. Although we tried to accommodate patient preferences by allowing them to choose the intervention modality (face-to-face group vs self-managed group with the BackFit app), the duration and intensity of our intervention seem insufficient to produce significant changes.

Furthermore, no significant differences between the groups or sessions in performance in terms of EEG activity during the Eriksen flanker task were found. Regarding cognitive performance, we found expected slower RTs in the incongruent trials, which confirmed the validity of this task to measure interference control, with greater cognitive resources needed to process stimuli in the incongruent condition. As the mean overall hit rate of this task was 98.93% (SD 0.14%), perhaps it was too effortless and not sensitive enough to observe changes produced by our educational and exercise-based intervention. Although current evidence backs cognitive improvements after aerobic exercise, we focused on a nonaerobic exercise–based intervention (including muscle strength exercises, motor control, relaxation, flexibility, and self-massage) to add novel evidence. In this regard, a previous study showed that a single session of aerobic exercise had no effect either on RTs or on brain activation in the Eriksen flanker task, but an explorative analysis revealed that RTs improved in both conditions after high-intensity exercise [69]. There is robust evidence in the literature of aerobic exercise being associated with structural and functional neuroplastic changes, partly mediated by epigenetic mechanisms, and improvements in cognitive functions and well-being [70]. It seems that in physical or metabolic training (eg, aerobic and strength), it is the intensity of training that enhances neuroplasticity (eg, reducing task-related activation of the superior and middle frontal cortex) and consequently improves cognition in a more global manner. Otherwise, in motor or neuromuscular training (eg, balance and coordination), it is the motor complexity that produces neuroplastic changes (eg, increasing activation in the inferior frontal gyrus and the superior parietal cortex, as well as in subcortical structures such as the thalamus and caudate body) and specific cognition improvements (eg, improving perceptual speed). Thus, according to current evidence, both intensity and motor complexity are important parameters to consider in the design of exercise interventions, which might have influenced our results.

### Limitations

Although the results are novel and interesting, there are several limitations in the design of this study that should be considered. The main limitation was not having a passive control group to compare both interventions. As mentioned previously, clinical practice guidelines recommend accommodating patient preferences in the design of such interventions. Therefore, to promote treatment adherence, participants were not randomly distributed; as a result, a risk of selection bias must be assumed. Because of the nature of the intervention, blinding of both researchers and participants was practically unattainable; however, this is also a bias that could compromise the internal validity of the study. Because of the exclusion of the data of 15% (9/59) of the participants, the study did not reach the planned sample size to achieve an adequate statistical power. Finally, we did not control for the use of caffeine before data collection and we did not restrict the use of medications, but there were no differences between the groups (Multimedia Appendix 2).

### Conclusions

Both intervention modalities (face-to-face group and self-managed group with the BackFit app) were equally effective at increasing beta activity at rest and located at the ACC, as well as at improving psychological functioning among patients with nonspecific CLBP. However, these results should be interpreted carefully because of the aforementioned limitations, which could compromise both internal and external validity of our study. The baseline pain and disability scores of our participants were clearly lower than those reported in previous studies; thus, they cannot be a representative sample of the population being studied. These limitations notwithstanding, to the best of our knowledge, this is the first study reporting brain changes in patients with CLBP after an mHealth intervention. Double-blinded randomized controlled studies with larger sample sizes are needed to increase the evidence for the efficacy of mHealth interventions in clinical practice for CLBP care. Furthermore, there is still conflicting evidence regarding the most adequate parameters for exercise prescription in chronic pain management, which must be considered in the design of novel exercise-based programs.

## Appendix

Multimedia Appendix 1 Nonsignificant results regarding electrocardiography data and pain sensitivity.

Multimedia Appendix 2 Percentage of causes of onset of pain, diagnosis, and surgery or invasive treatment received, as well as medication use.

## Abbreviations

ACC: anterior cingulate cortex
BA: Brodmann area
CBPT: cluster-based permutation test
CLBP: chronic low back pain
CONSORT-EHEALTH: Consolidated Standards of Reporting Trials of Electronic and Mobile Health Applications and Online Telehealth
ECG: electrocardiogram
EEG: electroencephalography
FABQ: Fear-Avoidance Beliefs Questionnaire
HF: high frequency
HR: heart rate
HRV: heart rate variability
LBP: low back pain
LF: low frequency
mHealth: mobile health
ODI: Oswestry Disability Index
PCS: Pain Catastrophizing Scale
POMS: Profile of Mood States
PPT: pressure pain threshold
RMSSD: root mean square of the successive differences
RT: reaction time
SDNN: SD of the normal-to-normal (R-R) intervals
TSK-11: Tampa Scale for Kinesiophobia
VLF: very low frequency
