# Supplementary material for: An Educational and Exercise Mobile Phone–Based Intervention to Elicit Electrophysiological Changes and to Improve Psychological Functioning in Adults With Nonspecific Chronic Low Back Pain (BackFit App): Nonrandomized Clinical Trial
Source: JMIR Mhealth Uhealth. 2022 Mar 15;10(3):e29171. doi: 10.2196/29171 (PMC8965676; doi:10.2196/29171)
Supplement: Multimedia Appendix 1 [file mhealth_v10i3e29171_app1.docx]

**Multimedia Appendix 1**. Nonsignificant results regarding electrocardiographic activity and pain sensitivity.

|  | **Before the intervention** | | **After the intervention** | | ***Group*** | ***Session*** | ***Session x group*** |
| --- | --- | --- | --- | --- | --- | --- | --- |
|  | **Face-to-face (n=27)**  **Mean (SD)** | **Self-managed (n=23)**  **Mean (SD)** | **Face-to-face (n=27)**  **Mean (SD)** | **Self-managed (n=23)**  **Mean (SD)** | ***P*** | ***P*** | ***P*** |
| **ECG activity**^a^ |  |  |  |  |  |  |  |
| HR | 70.00 (8.48) | 70.25 (10.39) | 70.06 (7.63) | 69.58 (11.30) | .962 | .788 | .750 |
| SDNN | 3.31 (0.39) | 3.51 (0.65) | 3.35 (0.35) | 3.62 (0.66) | .084 | .234 | .601 |
| RMSSD | 3.21 (0.53) | 3.42 (0.75) | 3.21 (0.53) | 3.48 (0.89) | .161 | .732 | .762 |
| VLF | 3.54 (1.11) | 4.03 (1.22) | 3.82 (0.99) | 4.32 (1.22) | .075 | .105 | .962 |
| LF | 5.89 (0.76) | 6.19 (1.40) | 5.99 (0.88) | 6.52 (1.26) | .144 | .141 | .434 |
| HF | 5.42 (1.03) | 5.81 (1.39) | 5.33 (1.10) | 5.84 (1.77) | .178 | .879 | .769 |
| **Pain sensitivity** |  |  |  |  |  |  |  |
| PPT lumbar (0-5 kg/cm^2^) | 3.49 (1.17) | 3.66 (1.23) | 3.23 (1.20) | 3.39 (1.18) | .602 | .060 | .966 |
| Pain rating lumbar (0-10) | 3.01 (1.95) | 2.29 (1.68) | 2.68 (1.82) | 2.32 (1.64) | .243 | .497 | .413 |
| PPT forefinger (0-5 kg/cm^2^) | 3.91 (1.01) | 4.13 (1.02) | 3.69 (1.19) | 4.07 (0.96) | .258 | .056 | .632 |
| Pain rating forefinger (0-10) | 2.25 (2.00) | 1.65 (1.42) | 1.95 (1.52) | 1.55 (1.55) | .195 | .492 | .430 |

ªAll these metrics, except for the mean HR, were transformed in a Napierian logarithmic scale before the statistical analyses.

Abbreviations: HR=heart rate; SDNN=standard deviations of the NN (R-R) intervals; RMSSD=root mean square of the successive differences; VLF=very low frequency; LF=low frequency; HF=high frequency; PPT=pressure pain threshold.
