# Supplementary material for: An Educational and Exercise Mobile Phone–Based Intervention to Elicit Electrophysiological Changes and to Improve Psychological Functioning in Adults With Nonspecific Chronic Low Back Pain (BackFit App): Nonrandomized Clinical Trial
Source: JMIR Mhealth Uhealth. 2022 Mar 15;10(3):e29171. doi: 10.2196/29171 (PMC8965676; doi:10.2196/29171)
Supplement: Multimedia Appendix 2 [file mhealth_v10i3e29171_app2.docx]

**Multimedia Appendix 2**. Percentage of causes of onset of pain, diagnosis, and surgery or invasive treatment received, as well as medication use.

|  | **Face-to-face (n=27), %** | **Self-managed (n=23), %** |
| --- | --- | --- |
| **Causes of onset of pain^1^** |  |  |
| Work accident | 3 (11.11%) | 3 (13.04%) |
| Traffic accident | 0 | 0 |
| Illness | 0 | 2 (8.69%) |
| Surgery | 0 | 0 |
| Spontaneous | 13 (48.15%) | 8 (34.78%) |
| Others | 7 (25.93%) | 7 (30.43%) |
| Not specified | 4 (14.81%) | 3 (13.04%) |
| **Diagnoses^2^**  **(total number of diagnoses)** | **21** | **9** |
| Hernia | 6 (28.57%) | 4 (44.44%) |
| Protrusion | 2 (9.52%) | 1 (11.11%) |
| Low back pain | 2 (9.52%) | 1 (11.11%) |
| Osteopenia | 1 (4.76%) | 0 |
| Contractures | 0 | 1 (11.11%) |
| Fracture | 1 (4.76%) | 0 |
| Nerve root symptoms | 1 (4.76%) | 1 (11.11%) |
| Degenerative pathology | 2 (9.52%) | 0 |
| Inflammation | 1 (4.76%) | 0 |
| Facet Syndrome | 1 (4.76%) | 0 |
| Anterolisthesis | 2 (9.52%) | 1 (11.11%) |
| Changes in sagittal curve | 1 (4.76%) | 0 |
| Scoliosis | 1 (4.76%) | 0 |
| **Surgery/invasive treatment^3^** | **3** | **3** |
| Infiltrations | 1 (33.33%) | 1 (33.33%) |
| Rhizolysis | 1 (33.33%) | 1 (33.33%) |
| Spine surgery | 1 (33.33%) | 1 (33.33%) |
| **Medication^4^ (number of prescriptions)** | **45** | **30** |
| Analgesics | 7 (15.55%) | 6 (20%) |
| Non-steroidal anti-inflammatory   drugs | 12 (26.66%) | 11 (36.66%) |
| Antidepressants | 3 (6.66%) | 1 (3.33%) |
| Proton-pump inhibitor | 1 (2.22%) | 2 (6.66%) |
| Antivertiginous | 0 | 1 (3.33%) |
| Fibrates | 1 (2.22%) | 0 |
| Antihistamines | 2 (4.44%) | 0 |
| Supplements | 0 | 0 |
| Cannabinoids | 0 | 1 (3.33%) |
| Triptans (migraine) | 1 (2.22%) | 1 (3.33%) |
| Anxiolytics | 1 (2.22%) | 0 |
| Muscle relaxant | 4 (8.88%) | 3 (10%) |
| Angiotensin II receptor antagonists | 0 | 1 (3.33%) |
| Beta-blockers | 0 | 1 (3.33%) |
| Benzodiazepines | 3 (6.66%) | 1 (3.33%) |
| Thyroid drugs | 1 (2.22%) | 1 (3.33%) |
| Ulcerative colitis drugs | 1 (2.22%) | 0 |
| Contraceptives | 1 (2.22%) | 0 |
| Hypnotics | 1 (2.22%) | 0 |
| Oral antidiabetics | 1 (2.22%) | 0 |
| Antihypertensive | 2 (4.44%) | 0 |
| Statins | 2 (4.44%) | 0 |
| Corticosteroids/Bronchodilators | 1 (2.22%) | 0 |

^1^n=43 participants (86%) reported a cause of onset of pain (Face-to-face: n=23, 46%; Self-managed: n=20, 40%). ^2^ n=18 participants (36%) reported having received one diagnose for their pain. From those, n=8 received two diagnoses (16%; Face-to-face: n=6, 75%) and n=4 received three (8%; Face-to-face: n=4, 100%). ^3^ n=6 participants (12%) reported having undergone an invasive treatment to relieve their pain (Face-to-face: n=3, 50%). In total, only n=2 participants reported having undergone infiltrations (33.33%; Face-to-face: n=1, 33.33%), rhyzolisis (33.33%; Face-to-face: n=1, 33.33%) or spine surgery (33.33%; Face-to-face: n=1, 33.33%). In total, 30 diagnoses were given to our sample (Face-to-face: n=21, 70%). The most prevalent diagnostics were hernias (n=10, 33.33), followed by protrusion (n=3, 10%), low back pain (n=3, 10%) and anterolisthesis (n=3, 10%). ^4^ n=31 participants (62%) reported consuming one drug (Face-to-face: n=18, 58,04%; Self-managed: n=13, 41,94%). From those, n=22 (44%) were consuming two drugs (Face-to-face: n=14, 63.64%), n=11 were consuming three (22%; Face-to-face: n=5, 45.45%), n=5 were consuming four (10%; Face-to-face: n=3, 60%), n=4 were consuming five (8%; Face-to-face: n=3, 75%) and n=2 were consuming six (4%; Face-to-face: n=2, 100%). In total, 75 drugs were prescribed in our sample (Face-to-face: 45; App: 30). The most prescribed drugs in our sample were non-steroidal anti-inflammatory drugs (30.6%), followed by analgesics (17.33%) and muscle relaxants (9.33%). This proportion was maintained at the face-to-face (26.66%, 15.55% and 8.88%, respectively) and the self-managed groups (36.66%, 20% and 10%, respectively); n=19 participants (38%) did not report taking drugs.
